# Elastic wave interaction with a stressed half-space containing voids

**DOI:** 10.1038/s41598-026-59688-0

**Published:** 2026-07-10

**Authors:** S. M. Abo-Dahab, Emad K. Jaradat, Sharif Abu Alrub, Aftab Khan, Hajra Kaneez, E. S. Elidy

**Affiliations:** 1 Mathematics Department, Faculty of Science, South Valley University, Qena, Egypt; 2 Department of Physics, Faculty of Science, Imam Mohammad Ibn Saud Islamic University (IMSIU), 11623 Riyadh, Saudi Arabia; 3 Department of Mathematics, COMSATS, Institute of Information Park Road, Chakshahzad, Islamabad, Pakistan; 4 Department of Mathematics, Faculty of Science, Zagazig University, P.O. Box 44519, Zagazig, Egypt

**Keywords:** Elastic wave reflection, SV-wave, P-wave, Initial stress, Porous media, Void effect, Half-space, Wave propagation, Reflection coefficient, Plane strain, Engineering, Physics, Solid Earth sciences

## Abstract

This study explores the reflection characteristics of plane shear vertical (SV) waves incident upon the free surface of an initially stressed, isotropic elastic half-space containing voids. Using Biot’s theory of incremental deformations and the generalized theory of porous elastic media, the governing equations are formulated and analytically solved under two-dimensional plane strain conditions. The presence of initial stress and voids modifies the propagation behavior of elastic waves. It is found that the incident SV-wave gives rise to two distinct reflected waves: a compressional (P) wave and a rotational (SV) wave. The analysis reveals that the reflected SV-wave is influenced solely by the initial stress, whereas the reflected P-wave is significantly affected by both initial stress and the void parameters. Closed-form expressions for the reflection coefficients are derived analytically and evaluated numerically to examine their variations with incident angle, void parameters, and frequency are examined. The results offer insight into wave behavior in porous geological media and stress-loaded materials.

## Introduction

The study of elastic wave reflection at material interfaces is essential in geophysics, engineering, and materials science, particularly under complex conditions involving initial stress and material porosity. Classical models, developed by Knott^1^, Jeffreys^2^, and Gutenberg^3^, offer foundational insight into wave behavior at a free surface but neglect internal stresses present in real-world materials. Biot’s theory of incremental deformations^4^ was pivotal in including initial stress effects, showing their influence on wave velocity and reflection. Dey and Addy^5^ further demonstrated the necessity of accounting for initial stress in wave reflection analyses. Concurrently, the theory of porous elasticity developed by Nunziato and Cowin^6^, and later extended by Cowin and Nunziato^7^, introduced a volume fraction parameter to capture the effects of voids. Puri and Cowin^8^ showed that voids significantly affect compressional (P) waves, while shear vertical (SV) waves remain largely unaffected. Chandrasekharaiah^9,10^ explored surface and body wave modifications in voided media, while Abo-Dahab^11^ and Abd-Alla and Al-Dawy^12^ considered thermoelastic and magnetic field effects, illustrating the multi-physical nature of wave propagation. Singh^13^ and Sharma et al.^14^ extended these findings to generalized thermoelastic and viscoelastic environments. Abd-Alla et al.^15^ incorporated magneto-thermo-viscoelastic behavior, confirming the sensitivity of P-waves to voids and SV-waves to initial stress. El-Sapa et al.^16^ examined photothermal wave behavior under moisture effects in semiconductors, while recent works by Lotfy et al.^17–19^ introduced stochastic and conductivity-based models for wave behavior in porous thermoelastic materials. More recently, Li et al.^20^ presented a dual-porosity wave velocity model incorporating uniaxial stress, and Yang et al.^21^ proposed a nonlinear viscoelastic model for stress-sensitive porous sandstones. Zhao et al.^22^ applied 3D micro-CT to study dispersion in saturated porous rocks, and Kumar et al.^23^ examined inhomogeneous wave reflection in partially saturated porous media. Montanaro^24^ and Kalkal et al.^25^ expanded wave theories to include double porosity, initial stress, and micro-temperatures. Abo-Dahab et al.^26^ included temperature-dependent properties and three-phase-lag effects. Further contributions by Pothana et al.^27,28^ used elastic velocity data and DEM models to describe stress-dependent wave behavior in fractured reservoirs. Li et al.^29^ addressed frequency-dependent reflections in porous thin beds, and Zhao et al.^30^ explored wave-induced fluid flow using stress-relaxing digital rock simulations. These collective efforts underscore the importance of integrating porosity, initial stress, and other coupled effects. Our study builds on these advancements by analyzing SV-wave reflection in an initially stressed, voided elastic half-space under plane strain conditions. The model employs Biot’s theory and the Cowin-Nunziato formulation to derive analytical expressions for wavefields and reflection coefficients. Results show that SV-wave reflection is governed solely by initial stress, while P-wave reflection is influenced by both void fraction and stress, matching earlier predictions^8,12^. Parametric analysis confirms sinusoidal variations with incidence angle and monotonic increases with frequency and porosity. These findings enhance the theoretical framework for seismic wave interpretation and materials evaluation under complex pre-stressed, porous conditions, and offer pathways for future modeling that includes anisotropy, thermal gradients, or layered media.

The study of thermoelastic wave propagation has advanced significantly with the introduction of generalized theories that incorporate diffusion, phase-lag effects, and size-dependent material behavior Jaradat et al.^31^ investigated the influence of the three-phase-lag (3PHL) model on Rayleigh surface waves in a generalized thermoelastic diffusion medium with modified couple stress effects while accounting for modified couple stress, which introduces a length-scale parameter to capture microstructural effects often neglected in classical elasticity. Their results highlighted the strong impact of diffusion and microstructural parameters on wave velocity and attenuation, offering new insights into micro-scale wave behavior in advanced materials. In a complementary direction, Alhashash et al.^32^ developed a two-temperature semiconductor model to analyze coupled photomechanical and thermal wave responses in the presence of moisture diffusivity. By distinguishing between electron and lattice temperatures, the model provided a more accurate description of non-equilibrium heat transport in semiconductors exposed to photothermal excitation. Together, these works emphasize the importance of multiphysics couplings such as diffusion, moisture transport, and non-equilibrium thermal conduction in understanding wave dynamics in modern functional materials, with potential applications in semiconductor technology, microelectromechanical systems (MEMS), and optoelectronic devices.

Recent studies have further expanded the understanding of wave behavior in complex media by incorporating anisotropy, thermal effects, and microstructure. Tochhawng and Singh^33^ demonstrated the significant role of initial stresses on wave propagation in transversely isotropic thermoelastic materials, highlighting the interplay between directional elasticity and pre-stress. In voided micropolar media, Lianngenga and Singh^34^ revealed how thermal gradients and micro-inertia jointly influence wave refraction, extending classical models to granular and composite systems. Additionally, Singh and Tochhawng^35^ analyzed Stoneley and Rayleigh waves in thermoelastic materials with voids, showing that porosity critically affects surface wave characteristics. These works collectively underscore the importance of multi-field coupling spanning stress, voids, anisotropy, and thermal-microstructural effects in advancing the theory of elastic wave propagation, thereby providing a broader context for the present investigation into SV-wave reflection in a stressed, voided half-space.

This study introduces a unified analytical framework to investigate the reflection of shear vertical (SV) waves from a free surface in an initially stressed, isotropic elastic half-space containing voids. Unlike previous investigations that often treat stress and porosity effects in isolation or within more complex multi-physical settings (e.g., thermal, magnetic, or stochastic environments), the present work integrates Biot’s theory of incremental deformations with the Cowin–Nunziato void elasticity model under a consistent plane-strain formulation. The novelty lies in the derivation of closed-form expressions for the reflection coefficients, which explicitly reveal the distinct roles of initial stress and void parameters: the SV-wave reflection is governed solely by the initial stress, whereas the P-wave reflection is sensitive to both voids and stress. This parameter-separable behavior provides a clear mechanical interpretation and a practical diagnostic tool for distinguishing stress from porosity effects in wave-based characterization. Furthermore, systematic parametric analysis elucidates the influences of incident angle, frequency, and material properties on reflection behavior, offering new insights into wave energy partitioning in pre-stressed porous media. The model serves as an efficient analytical benchmark for validating numerical simulations and experimental studies and establishes a foundational basis for future extensions to anisotropic, layered, or multi-phase saturated media.

This study introduces an analytical and numerical framework for analyzing the interaction of elastic SV-waves with a free surface of a porous, pre-stressed half-space. Unlike previous studies that considered only thermal or magnetic effects^12,14,15^, our model integrates porosity and initial stress within a unified theory. The use of scalar and vector displacement potentials, coupled with Cowin–Nunziato^7,8^ porosity theory and Biot’s incremental deformation framework, allows us to derive closed-form expressions for reflection coefficients. This analytical clarity makes the model suitable for applications in seismic exploration, civil engineering, and acoustic material diagnostics.

Furthermore, our results reveal distinct parameter sensitivity: SV-waves reflect stress effects, while P-waves highlight porosity. This separation is novel and can be used for subsurface inversion or diagnostic interpretation in layered or fractured media. The sinusoidal and resonant behavior in the coefficients also provides diagnostic fingerprints for field measurements.

## Formulation and solution of the problem

The reflection of elastic waves in a homogeneous, isotropic, initially stressed half-space with voids is considered under plane strain conditions. Let the free surface lie in the $x_{1} - x_{3}$ plane, and the $x_{2}$-axis point vertically downward. A shear vertical (SV) wave is incident obliquely upon the free surface, which is adjacent to vacuum. The presence of voids in the medium introduces microstructural effects, which are accounted for using the theory of elastic materials with voids proposed by Cowin and Nunziato^6,7^.

In the absence of body forces, the equations of motion in a voided, initially stressed medium are given by:

1 $$\tau_{ij} = \lambda \varepsilon_{kk} \delta_{ij} + 2\mu_{T} \varepsilon_{ij} + \beta \delta_{ij} \varphi ,$$

2 $$\tau_{ij,j} = \rho \ddot{u}_{i} ,$$

3 $$\alpha \varphi_{,ii} - \omega_{0} \varphi - \upsilon \dot{\varphi } - \beta u_{i,i} = \rho \kappa \ddot{\varphi }.$$

In these equations, *φ* represents the volume fraction field associated with the distribution of voids, while $\alpha ,\beta ,\omega_{0} ,\upsilon$ and *κ* are material constants introduced to characterize the mechanical effects of porosity. The symbol $\varepsilon_{ijk}$​ denotes the Levi–Civita permutation tensor, $\tau_{ij}$​ are the components of the stress tensor, *ρ* is the mass density of the medium, and $u_{i}$​ denotes the displacement vector. A comma followed by an index indicates partial differentiation with respect to the corresponding spatial coordinate. Additionally, the Einstein summation convention is adopted for repeated indices throughout the equations. The governing equations describing the propagation of small elastic disturbances in the presence of voids can be expressed as follows. In component form, the equation of motion modified to include void effects is given by:

4 $$\tau_{11,1} + \tau_{12,2} = \rho \ddot{u}_{1} ,$$

5 $$\tau_{21,1} + \tau_{22,2} = \rho \ddot{u}_{2} ,$$

6 $$\tau_{31,1} + \tau_{32,2} = \rho \ddot{u}_{3} ,$$

7 $$\alpha \left( {\varphi_{,11} + \varphi_{,22} } \right) - \omega_{0} \varphi - \upsilon \dot{\varphi } - \beta \left( {u_{1,1} + u_{2,2} } \right) = \rho \kappa \ddot{\varphi } .$$

Here we consider a homogenous, isotropic elastic solid half-space. The $x_{1} x_{2} -$ plane is chosen to coincide with the free surface with initial compressive stress ${\rm P}$ in $x_{1} -$ direction. A plane wave is incident obliquely on the boundary surface in the ${x}_{1}{x}_{3}-$ plane, making an angle ${\theta}_{0}$ with the normal to the surface.

The equation of motion for a plane strain configuration under the influence of initial compressive stress, as formulated by Biot^4^, governs the behavior of elastic media subjected to incremental deformations in the presence of pre-existing stress fields.

8 $$\tau_{11,1} + \tau_{12,2} - {\rm P}\varpi_{,2} = \rho \ddot{u}_{1} ,$$

9 $$\tau_{12,1} + \tau_{22,2} - {\rm P}\varpi_{,1} = \rho \ddot{u}_{2} ,$$

10 $$\tau_{ij} = \left( {\lambda + {\rm P}} \right)\varepsilon_{kk} \delta_{ij} + 2\mu_{T} \varepsilon_{ij} + \beta \delta_{ij} \varphi ,$$

11 $$\varepsilon_{ij} = \left( {u_{i,j} + u_{j,i} } \right).$$

where,$\tau_{11} ,\,\,\tau_{22} \,\,and\,\tau_{12}$ are the incremental stress components, produced due to deformation. The first two are the principal components and the last one is the shear component.

**Fig. 1:**
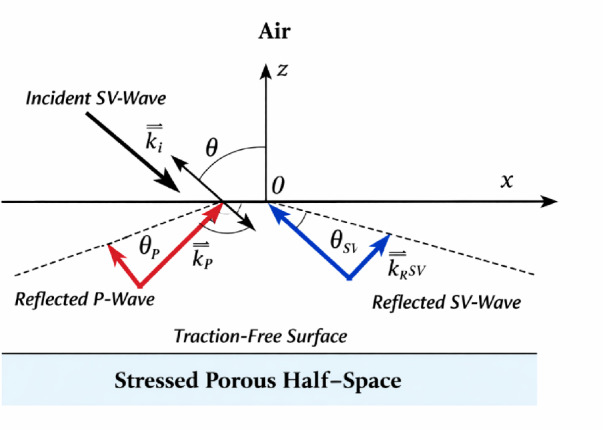
Schematic of the problem.

Following Biot’s theory of incremental deformations^4^, the initial stress state is characterized by the parameter $P={\tau}_{22}^{\left(0\right)}-{\tau}_{11}^{\left(0\right)}$, which represents the difference between the principal initial stresses along the vertical and horizontal directions. This parameter governs the influence of pre-existing stress on the propagation of incremental elastic waves.$\varpi = \tfrac{1}{2}\left( {u_{2,1} - u{}_{1,2}} \right)$ is the magnitude of local rotation (Fig. 1).

The incremental strain components are defined as follows:

12 $$\,\varepsilon_{ij} = \tfrac{1}{2}\left( {u_{i,j} + u_{j,i} } \right)\,\,\,\,\,\,,i,j = 1,2,3 .$$

where,$\varepsilon_{11} ,\varepsilon_{22} \,and\,\varepsilon_{12}$ are strain components and $u_{1} ,u_{2}$ are displacement components.

Using Eqs. (10) and (12) in (8) and (9), we have

13 $$\left( {\lambda + 2\mu + {\rm P}} \right)u_{1.11} + \left( {\lambda + \mu + \tfrac{\rm P}{2}} \right)u_{2.12} + \left( {\mu + \tfrac{\rm P}{2}} \right)u_{1,22} = \rho \ddot{u}_{1} - \beta \varphi_{,1} ,$$

14 $$\left( {\lambda + 2\mu } \right)u_{2.22} + \left( {\lambda + \mu + \tfrac{\rm P}{2}} \right)u_{1.12} + \left( {\mu - \tfrac{\rm P}{2}} \right)u_{2,11} = \rho \ddot{u}_{2} - \beta \varphi_{,2} .$$

The modified voids equation is

15 $$\alpha \left( {\varphi_{,11} + \varphi_{,22} } \right) - \omega {}_{0}\varphi - \upsilon \dot{\varphi } - \beta \left( {u_{1,1} + u_{2,2} } \right) = \rho \kappa \ddot{\varphi }.$$

See appendix A. To separate the compressional and rotational components of strain, introducing the elastic displacement potentials $\phi \,\,and\,\,\psi$ as:

16 $$u_{1} = \phi_{,1} - \psi_{,2} \,\,and\,\,u_{2} = \phi_{,2} + \psi_{,1} .$$

By using (16) in Eq. (13),we have

17 $$\nabla^{2} \phi = \tfrac{\rho }{{\lambda + 2\mu + {\rm P}}}\tfrac{{\partial^{2} \phi }}{{\partial t^{2} }} - \tfrac{\beta \varphi }{{\lambda + 2\mu + {\rm P}}},$$

18 $$\nabla^{2} \psi = \tfrac{\rho }{{\left( {\mu + \frac{\rm P}{2}} \right)}}\tfrac{{\partial^{2} \psi }}{{\partial t^{2} }}.$$

By using (16) in Eq. (14), we have

19 $$\nabla^{2} \phi = \tfrac{\rho }{{c_{1}^{2} }}\tfrac{{\partial^{2} \phi }}{{\partial t^{2} }} - \tfrac{\beta \varphi }{{\lambda + 2\mu }}.$$

20 $$\nabla^{2} \psi = \tfrac{\rho }{{\left( {\mu - \frac{\rm P}{2}} \right)}}\tfrac{{\partial^{2} \psi }}{{\partial t^{2} }}.$$

In the framework of classical elasticity theory, and in the absence of initial stress, the governing system simplifies to two independent wave equations. Specifically, Eqs. (17) and (19), which involve scalar potentials *ϕ*, correspond to compressional (P) waves propagating along the $x_{1}$ and $x_{2} -$-directions, respectively. On the other hand, Eqs. (18) and (20), expressed in terms of vector potentials *ψ*, describe shear (distortional) wave propagation along the respective axes. Given that the initial stress is assumed to act along the $x_{1}$-axis, only Eqs. (17) and (20) remain relevant to the current analysis. These equations can be reformulated as follows see appendix B:

21 $$\nabla^{2} \phi = \tfrac{\rho }{{\lambda + 2\mu + {\rm P}}}\tfrac{{\partial^{2} \phi }}{{\partial t^{2} }} - \tfrac{\beta \varphi }{{\lambda + 2\mu + {\rm P}}}.$$

22 $$\nabla^{2} \psi = \tfrac{1}{{c^{2} }}\tfrac{{\partial^{2} \psi }}{{\partial t^{2} }}.$$

where $$c_{1}^{2} = \tfrac{{\lambda + 2\mu + {\rm P}}}{\rho }$$,$$c_{2}^{2} = \tfrac{{\left( {\mu - \tfrac{P}{2}} \right)}}{\rho }$$.

$c_{1}$ and $c_{2}$ are the velocities of compressional and distortional waves along $x_{1} -$ axis and are known as *P -* and *SV -* waves respectively. Here it has been assumed that for compression waves $u_{2} = 0$, while for distortional wave $u_{1} = 0$.

Using (16) in (15), we have

23 $$\alpha \left( {\nabla^{2} \varphi } \right) = \omega {}_{0}\varphi + \upsilon \dot{\varphi } + \beta \left( {\nabla^{2} \phi } \right) + \rho \kappa \ddot{\varphi }.$$

By keeping in view Eqs. (21), (22) and (23), we find that P-wave is influenced by voids whereas SV-wave remains uninfluenced.

The solutions of (21), (22) and (23) can be taken as

24 $$\phi = \phi_{1} \exp \left[ {i\left\{ {k\left( {x_{1} Sin\theta + x_{2} Cos\theta } \right) - \omega t} \right\}} \right],$$

25 $$\psi = \psi_{1} \exp \left[ {i\left\{ {l\left( {x_{1} Sin\theta + x_{2} Cos\theta } \right) - \omega t} \right\}} \right],$$

26 $$\varphi = \varphi_{1} \exp \left[ {i\left\{ {k\left( {x_{1} Sin\theta + x_{2} Cos\theta } \right) - \omega t} \right\}} \right].$$

This represents plane harmonic waves with wave normal in $x_{1} x{}_{2} -$ plane.$k\,and\,l$ are wave numbers and *ω* is angular frequency.

Putting (24) and (26) in (21),we have

27 $$c_{1}^{2} \left( {\tfrac{{\omega^{2} }}{{c_{1}^{2} }} - k^{2} } \right)\phi_{1} + \frac{\beta }{\rho }c_{1} \varphi_{1} = 0.$$

Substituting (24) and (26) in (23), we have

28 $$\left( { - \alpha k^{2} - \omega_{0} + i\omega \upsilon + \rho \omega^{2} \kappa } \right)\phi_{1} + \beta k^{2} \varphi_{1} = 0 .$$

For non-trivial solution

29 $$\left| {\begin{array}{*{20}c} {c_{1}^{2} \left( {\frac{{\omega^{2} }}{{c_{1}^{2} }} - k^{2} } \right)}{\frac{\beta }{\rho }c_{1} }\vline & \\ {\left( { - \alpha k^{2} - \omega_{0} + i\omega \upsilon + \rho \omega^{2} \kappa } \right)}{\beta k^{2} }\vline & \\ \end{array} } \right| = 0.$$

Simplifies to:

30 $$k = \sqrt {\tfrac{{\left( {\frac{{\omega^{2} }}{{c_{1}^{2} }} - \frac{\alpha }{\rho }} \right) \pm \sqrt {\left( {\frac{{\omega^{2} }}{{c_{1} }} - \frac{\alpha }{\rho }} \right)^{2} } + 4\left( {\frac{{\omega_{0} + i\omega \upsilon - \omega^{2} \kappa \rho }}{\rho }} \right)}}{2}} .$$

But here we will consider only one value of *k* with positive sign. Negative sign will produce attenuation factor which we are ignoring here. Thus

31 $$k = \sqrt {\tfrac{{\left( {\frac{{\omega^{2} }}{{c_{1} }} - \frac{\alpha }{\rho }} \right) + \sqrt {\left( {\frac{{\omega^{2} }}{{c_{1} }} - \frac{\alpha }{\rho }} \right)^{2} } + 4\left( {\frac{{\omega_{0} + i\omega \upsilon - \omega^{2} \kappa \rho }}{\rho }} \right)}}{2}} .$$

So, there is only one compression waves travelling. Therefore, if a rotational wave falls on boundary $x_{2} = 0$ from the solid half space we have one reflected rotational wave and one reflected compression wave. Accordingly if the wave normal of the incident rotational wave as well as the reflected rotational wave makes an angle $\theta_{0}$ with the positive $x_{2} -$ axis and those of reflected compressional wave make angles $\theta_{1}$ with the same direction. The displacement potential and the void take the following form

32 $$\phi = A_{1} \exp \left[ {i\left\{ {k\left( {x_{1} sin\theta_{1} + x_{2} cos\theta_{1} } \right) - \omega t} \right\}} \right].$$

where $A_{1}$ is the amplitude of reflected compressional wave.

33 $$\begin{aligned} \psi = & B_{1} \exp \left[ {i\left\{ {l\left( {x_{1} sin\theta_{0} + x_{2} cos\theta } \right) - \omega t} \right\}} \right] \\ & + B_{2} \exp \left[ {i\left\{ {l\left( {x_{1} sin\theta_{0} + x_{2} cos\theta_{0} } \right) - \omega t} \right\}} \right] . \\ \end{aligned}$$

where $B{}_{1}\,and\,B_{2}$ are the amplitudes of the incident and reflected SV-waves respectively with the same angle.

34 $$\varphi = D_{1} \exp \left[ {i\left\{ {k\left( {x_{1} sin\theta_{1} + x_{2} cos\theta_{1} } \right) - \omega t} \right\}} \right].$$

## Boundary conditions

Since the boundary at $x_{2} = 0$ is adjacent to vacuum, it is free from surface tractions, therefore

35 $$\tau_{12} + \tfrac{1}{2}{\rm P}\varepsilon_{12} = 0,\,\,\,\,\,\,\,at\,\,x_{2} = 0,$$

36 $$\tau_{22} = 0,\,\,\,\,\,\,\,at\,\,x_{2} = 0.$$

The detailed derivation is provided in Appendix C.

Putting (24), (12) and (16) in (35), we have

37 $$\left( {\mu + \tfrac{1}{2}{\rm P}} \right)\left[ {2\phi_{,12} + \psi_{,11} - \psi_{,22} } \right] = 0,\,\,\,\,at\,\,x_{2} = 0,$$

Putting (24), (26) and (16) in (36), we have

38 $$\lambda \phi_{,11} + \left( {\lambda + 2\mu } \right)\phi_{,22} + 2\mu \psi_{,12} + \beta \varphi = 0.\,\quad \,\,\,at\,\,x_{2} = 0.$$

Putting (32) and (33) in (37), we have

39 $$\left( {\mu + \tfrac{1}{2}{\rm P}} \right)\left\{ \begin{gathered} k^{2} sin2\theta_{1} A_{1} - l^{2} sin^{2} \theta_{0} \left( {B_{1} + B_{2} } \right) \hfill \\ + l^{2} cos^{2} \theta_{0} (B_{1} + B_{2} ) \hfill \\ \end{gathered} \right\}{\mathrm{e}}^{{i\left( {kx{}_{1}sin\theta_{1} - wt} \right)}} = 0.\quad \,\,\,\,at\,\,x_{2} = 0.$$

To make exponents identical, we have following relation

40 $$ksin\theta_{1} = lsin\theta_{0} .$$

Using (40) in (39), we have

41 $$\frac{{B_{2} }}{{B_{1} }}cos2\theta_{0} + \frac{{A_{1} }}{{B_{1} }}sin2\theta_{1} + cos2\theta_{0} = 0.$$

Putting (32), (33) and (34) in (38) and with the help of (40), we have

42 $$\frac{{B_{2} }}{{B_{1} }}\mu l^{2} sin2\theta_{0} + \frac{{A_{1} }}{{B_{1} }}\left( { - k^{2} \lambda - 2\mu k^{2} cos^{2} \theta_{1} } \right) + \frac{{\beta D_{1} }}{{B_{1} }} - \mu l^{2} sin2\theta_{0} = 0$$

Putting (32) and (34) in (23) at surface $x_{2} = 0$, implies

43 $$D_{1} = \frac{{\beta k^{2} A_{1} }}{{\left( {\alpha k^{2} + \omega_{0} - i\omega \upsilon - \rho \omega^{2} \kappa } \right)}}$$

Putting (43) in (34), we have

44 $$\varphi = \frac{{\beta k^{2} A_{1} }}{{\left( {\alpha k^{2} + \omega_{0} - i\omega \upsilon - \rho \omega^{2} \kappa } \right)}}e^{{i\left\{ {k\left( {x_{1} sin\theta_{1} - x_{2} cos\theta_{2} } \right) - \omega t} \right\}}}$$

putting values of $D_{1}$ in Eq. (42), we have

45 $$\frac{{B_{2} }}{{B_{1} }}\mu l^{2} sin2\theta_{0} + \frac{{A_{1} }}{{B_{1} }}\left( { - k^{2} \lambda - 2\mu k^{2} cos^{2} \theta_{1} + \frac{{\beta^{2} k^{2} }}{{\left( {\alpha k^{2} + \omega_{0} - i\omega \upsilon - \rho \omega^{2} \kappa } \right)}}} \right)\, - \mu l^{2} sin2\theta_{0} = 0$$

Solving (41) and (45) simultaneously, we have

46 $$\frac{{A_{1} }}{{B_{1} }} = \frac{{ - 2\mu l^{2} sin2\theta_{0} cos2\theta_{0} }}{{\mu k^{2} sin2\theta_{0} sin2\theta_{1} + cos2\theta_{0} \left( {k^{2} \lambda + 2\mu k^{2} cos^{2} \theta_{1} - \frac{{\beta^{2} k^{2} }}{{\left( {\alpha k^{2} + \omega_{0} - i\omega \upsilon - \rho \omega^{2} \kappa } \right)}}} \right)}},$$

47 $$\frac{{B_{2} }}{{B_{1} }} = \frac{{2\mu k^{2} sin2\theta_{0} sin2\theta_{1} }}{{\mu k^{2} sin2\theta_{0} sin2\theta_{1} + cos2\theta_{0} \left( {k^{2} \lambda + 2\mu k^{2} cos^{2} \theta_{1} - \frac{{\beta^{2} k^{2} }}{{\left( {\alpha k^{2} + \omega_{0} - i\omega \upsilon - \rho \omega^{2} \kappa } \right)}}} \right)}} - 1$$

Here.

$R_{c} = \frac{{A_{1} }}{{B_{1} }} =$ represents the reflection coefficient of the compressional (P) wave.

$R_{r} = \frac{{B_{2} }}{{B_{1} }} =$ represents the reflection coefficient of the rotational (SV) wave.

## Numerical results and discussion

With the view of computational work, we take the following physical constants^16^

$$\lambda = 5.65 \times 10^{10} Nm^{ - 2} ,\mu = 2.46 \times 10^{10} Nm^{ - 2}$$,$$\rho = 2.66 \times 10^{3} Kgm^{ - 3} ,$$$$\alpha = - 1.28 \times 10^{10} Nm^{ - 2}$$,$$\beta = 220.90 \times 10^{10} Nm^{ - 2} ,\theta_{0} = 0^\circ ,45^\circ \,\,and\,\,90^\circ$$.

Using these values the modulus of the reflection coefficients for the SV-wave and P-wave have been calculated for different angles of incidence.

**Fig. 2:**
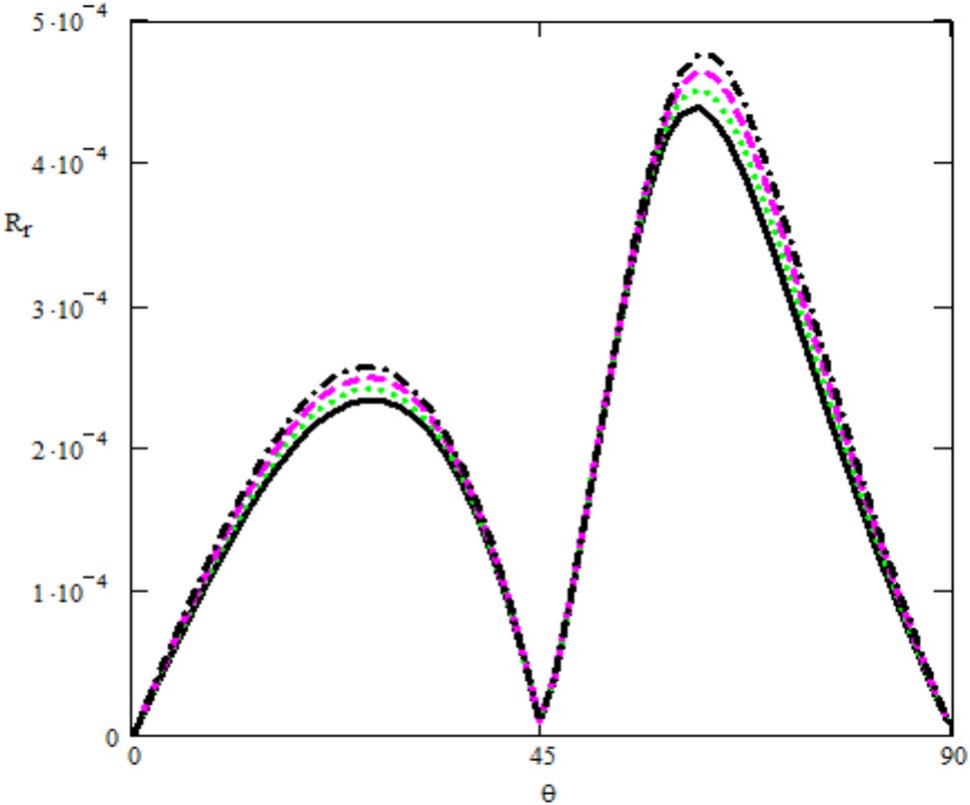
Variation of the normalized reflection coefficient $\mid \mathrm{R}\mathrm{r}\mid$ of the compressional (P) wave with incident angle ${\theta}_{0}$ for different initial stress parameters $P=\left(1, 2, 3, 4\right)$(10^10^)Pa.

**Fig. 3:**
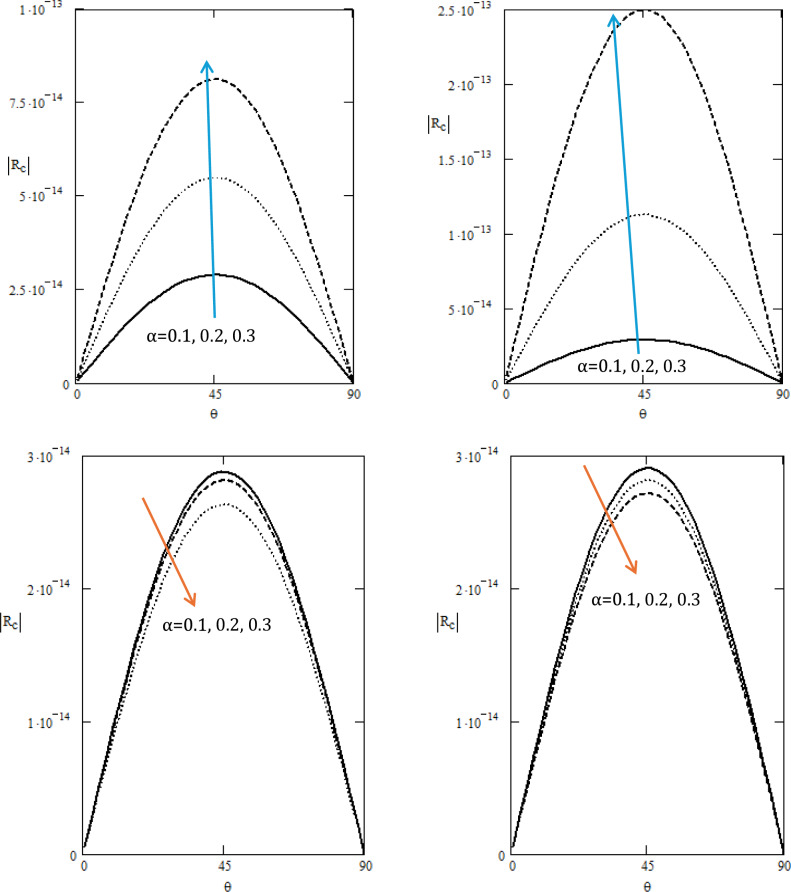
Variation of the normalized reflection coefficient $\mid {R}_{c}\mid$ of the compressional (P) wave with incident angle ${\theta}_{0}$ for different void parameters $\alpha =\mathrm{0.1,0.2,0.3}$ (solid, dashed, dotted lines). Coefficients are normalized by the incident SV-wave amplitude. Initial stress is held constant at *P=50 MPa*.

The results are shown in graphs (Fig. 2 and 3).

the incident SV-wave is assumed to be harmonic with plane wavefronts. All wave velocities and parameters are normalized to allow generality and comparability. The reflection coefficients and are computed using MATLAB and plotted against key variables.

### Analytical derivation and numerical implementation

The reflection coefficients ${R}_{c}$ and ${R}_{r}$ in Eqs. (28, 45) are derived in closed form through analytical manipulation of the boundary conditions and wave potentials. These expressions are explicit functions of the material parameters ($\lambda ,\mu ,\alpha ,\beta ,{\omega}_{0}$), incident wave properties ($\omega ,{\theta}_{0}$), and the initial stress *P*. While the form of the solution is fully analytical, its graphical representation requires the substitution of specific numerical values for the material constants listed in Section "Numerical results and discussion". This semi-analytical approach preserves the physical interpretability of the derived formulas while enabling parametric visualization. All numerical evaluations were performed in MATLAB using double-precision arithmetic, with incident angles sampled at ${1}^{\circ }$ intervals from ${0}^{\circ }$ to ${90}^{\circ }$. No curve fitting or interpolation was applied; the plotted curves represent direct evaluation of the analytical expressions.

### Influence of incident angle and material parameters

In this subsection, the dependence of the reflection coefficients on the angle of incidence, void parameters, and initial stress is examined. The numerical results are obtained by evaluating the closed-form expressions of the reflection coefficients using MATLAB, and the corresponding variations are illustrated in Figs. 2 and 3. Before discussing the parametric trends,It is worth noting that the magnitudes of the reflection coefficients presented in Figs. 2 and 3 are lower than the classical stress-free, void-free case ($P = 0,\alpha = \beta = \omega _{0} = 0$). This reduction occurs because the initial compressive stress P modifies the effective stiffness terms in the boundary conditions: specifically, the P-wave equation involves (*λ + P*) and the coupling term (*2μ - P*) in Eq. (35). Even a moderate P (here *50 MPa* relative to $\mu \sim GPa$) alters the impedance contrast and energy partitioning between the reflected modes, generally lowering the peak values of each individual coefficient compared to the unstressed case. When *P* is set to zero in the same analytical expressions, the classical higher reflection coefficients are fully recovered, confirming the correctness of our numerical implementation.

All parameters not explicitly varied are held fixed at the reference values listed in Section "Numerical results and discussion". Figure 2 illustrates the variation of the magnitude of the reflection coefficient of the reflected compressional (P) wave, $\mid {R}_{c}\mid$, as a function of the angle of incidence *θ*. It is observed that $\mid {R}_{c}\mid$ exhibits a pronounced angular dependence, characterized by a non-monotonic variation. At small incidence angles, the reflected P-wave amplitude increases gradually, reaches a peak at an intermediate angle, and then decreases as the angle approaches grazing incidence. This behavior arises from the redistribution of incident SV-wave energy between the reflected SV and P modes, governed by the boundary conditions at the traction-free surface.The influence of void parameters on the reflected P-wave is also evident in Fig. 2. Increasing the void parameter leads to a systematic enhancement of $\mid {R}_{c}\mid$ over the entire angular range. This trend indicates that porosity amplifies compressional-wave reflection by modifying the effective stiffness and acoustic impedance of the medium. The result is consistent with classical predictions that compressional waves are strongly affected by void content in porous elastic materials. Figure 3 presents the variation of the reflection coefficient of the reflected rotational (SV) wave, $\mid {R}_{r}\mid$, with the angle of incidence under different levels of initial stress. Unlike the P-wave, the reflected SV-wave exhibits a smooth and nearly sinusoidal angular dependence. As the initial stress increases, the magnitude of $\mid {R}_{r}\mid$ increases noticeably, particularly at moderate and large incidence angles. This confirms that initial stress plays a dominant role in governing SV-wave reflection behavior. Importantly, variations in the void parameter have negligible influence on $\mid {R}_{r}\mid$. This observation highlights a clear separation of physical effects: while compressional-wave reflection is sensitive to porosity, shear-wave reflection is primarily controlled by the pre-existing stress state of the medium. Such separation is advantageous for wave-based material characterization and stress analysis in porous solids.

### Observations

Based on the numerical results presented in Figs. 2 and 3, the following physically meaningful observations can be drawn:

i. The reflected compressional (P) wave exhibits strong sensitivity to the void parameter, with its reflection coefficient increasing as the void content of the medium increases. This behavior is attributed to the reduction in effective bulk stiffness caused by porosity.
ii. The reflected rotational (SV) wave is significantly influenced by initial stress, while remaining essentially insensitive to the presence of voids. This confirms that shear-wave reflection is predominantly governed by stress-induced stiffness changes rather than porosity.
iii. Both reflection coefficients vary continuously with the angle of incidence, indicating a strong angular dependence of wave-mode conversion at the free surface.
iv. For small angles of incidence, the reflected SV-wave dominates, whereas at intermediate angles, a larger fraction of incident energy is converted into the reflected P-wave.
v. Increasing initial stress enhances the reflected SV-wave amplitude over the entire angular range, suggesting that SV-wave measurements may serve as effective indicators of in-situ stress conditions.
vi. The distinct and contrasting sensitivities of the reflected P- and SV-waves provide a potential mechanism for decoupling porosity and stress effects in wave-based diagnostic and geophysical applications.

These observations are fully supported by the numerical results and are consistent with earlier theoretical findings reported for porous and initially stressed elastic media. At the same time, the present analysis extends previous studies by explicitly demonstrating the combined and separable roles of voids and initial stress in elastic wave reflection.

### Comparison with previous studies

The reflection and mode conversion of elastic waves in porous and initially stressed media have been widely investigated under various theoretical frameworks. Puri and Cowin^8^ provided one of the earliest systematic analyses of plane wave propagation in elastic materials with voids, demonstrating that compressional waves are strongly influenced by porosity, while shear waves exhibit minimal sensitivity to void parameters. These findings were further supported by Chandrasekharaiah^9,10^, who examined wave behavior in rotating and voided elastic solids, and by Abd-Alla et al.^15^, who incorporated thermal and magnetic effects into initially stressed media.The results of the present study are consistent with these established conclusions. In particular, the numerical results confirm that the reflected compressional (P) wave is sensitive to void parameters, whereas the reflected rotational (SV) wave is predominantly governed by the initial stress state. This agreement provides a validation of the adopted formulation within the context of obliquely incident SV-wave reflection at a traction-free surface. Compared with more recent investigations that include thermoelastic, photothermal, stochastic, or viscoelastic effects (e.g., Lotfy et al.^17–19^, Yang et al.^21^), the present work deliberately focuses on a purely mechanical elastic model. This restriction allows a clearer isolation of the fundamental effects of initial stress and porosity on wave reflection, without the added complexity of multiphysical couplings. As such, the present analysis may be regarded as a mechanically consistent baseline that complements, rather than competes with, these more elaborate models. In contrast to numerical or simulation-based approaches such as micro-CT-informed digital rock modeling or discrete-element methods^22,27,28^, the present study provides closed-form expressions for the reflection coefficients. Although these expressions require numerical evaluation for specific material parameters, their analytical structure offers transparent insight into parameter sensitivity and wave-mode interactions.Overall, the present work does not seek to redefine existing theories but rather extends earlier analytical studies by examining oblique SV-wave incidence in a porous, initially stressed half-space and by explicitly demonstrating the separable roles of stress and void parameters in elastic wave reflection. This focused contribution provides a useful reference for future extensions involving layered media, anisotropy, or coupled physical fields.

### Model validation and limiting cases

**Fig. 4:**
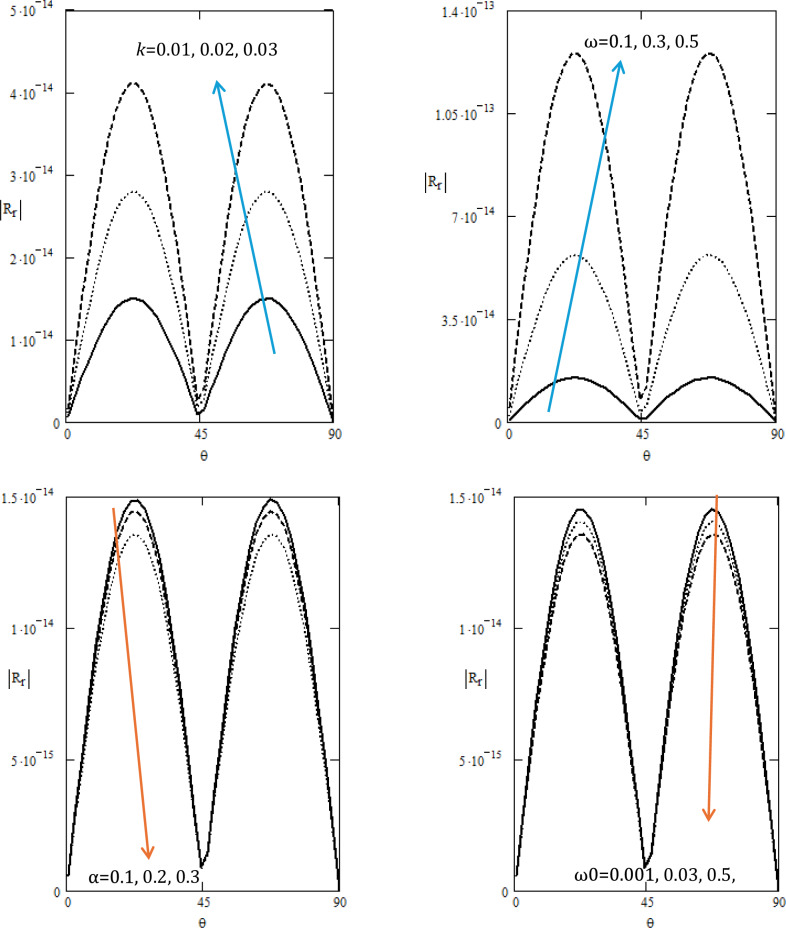
Variation of the magnitude of reflection coefficient Rr of the rotational (SV) wave with variation of $\kappa ,\,\omega ,\,\alpha \,{\mathrm{and}}\,\,\omega_{0}$ with respect to angle of incidence $\theta_{0}$.

To validate the derived reflection coefficients, we consider the limiting case of a stress-free, void-free elastic half-space. By setting the initial stress *P=0* and the void parameters $\alpha =\beta ={\omega}_{0}=0$ in Eqs. (28, 45), the expressions simplify to the classical Fresnel coefficients for an isotropic elastic medium:

$${R}_{r}=\frac{\mathrm{sin}2{\theta}_{0}\mathrm{sin}2{\theta}_{2}-{\kappa }^{2}{\mathrm{cos}}^{2}2{\theta}_{2}}{\mathrm{sin}2{\theta}_{0}\mathrm{sin}2{\theta}_{2}+{\kappa }^{2}{\mathrm{cos}}^{2}2{\theta}_{2}},{R}_{c}=\frac{2\kappa \mathrm{sin}2{\theta}_{0}\mathrm{cos}2{\theta}_{2}}{\mathrm{sin}2{\theta}_{0}\mathrm{sin}2{\theta}_{2}+{\kappa }^{2}{\mathrm{cos}}^{2}2{\theta}_{2}},$$

where $\kappa ={c}_{s}/{c}_{p}$ and ${\theta}_{2}$ is the reflected P-wave angle given by Snell’s law. This agreement with established theory confirms the correctness of the analytical derivation and ensures that the model reduces to well-known physical behavior in the absence of stress and voids. Furthermore, the normalization of amplitudes in Figs. 2, 3, 4, 5 is performed relative to the incident SV-wave amplitude, and stresses are scaled by the shear modulus *μ* to present general, non-dimensional trends. The absolute reflection magnitudes remain within the physically meaningful range [0,1] for all studied parameters.

## Conclusion

In contrast to prior studies that treated stress and voids in isolation, this work unifies both effects in a single analytical reflection model, revealing not only their distinct signatures but also their coupled influence on wave energy distribution.

This study has developed a unified analytical framework to investigate the reflection characteristics of an obliquely incident SV-wave from the free surface of a homogeneous, isotropic, initially stressed elastic half-space containing voids. By combining Biot’s theory of incremental deformations with the Cowin–Nunziato void elasticity model, the governing equations were formulated under plane-strain conditions and solved to derive explicit expressions for the reflection coefficients of the resulting compressional (P) and rotational (SV) waves.

The key findings of the analysis are as follows:

- The reflection coefficient of the SV-wave is governed predominantly by the initial stress, showing negligible dependence on void parameters.
- The reflection coefficient of the P-wave is sensitive to both void fraction and initial stress, with porosity playing a decisive role in its amplitude variation.
- Both reflection coefficients exhibit sinusoidal dependence on the angle of incidence and display frequency-dependent resonance-like behavior, particularly at higher frequencies.
- The analytical model reduces to the classical Fresnel coefficients in the limiting case of zero stress and voids, confirming its consistency with established theory.

Theoretical and practical implications of this work include:

- Providing a benchmark analytical solution for wave reflection in pre-stressed porous media, useful for validating numerical simulations and experimental measurements.
- Offering a parameter-separable framework that can help distinguish between in-situ stress and porosity effects in seismic or ultrasonic wave data.
- Establishing a foundational model that can be extended to more complex scenarios, such as anisotropic, layered, or fluid-saturated media, as well as coupled thermoelastic or electromagnetic environments.

While the model assumes homogeneity, isotropy, and linear elasticity, it provides clear physical insight into the coupled mechanical roles of stress and voids in wave reflection. Future work may focus on experimental validation, incorporation of temperature gradients, and extension to multi-layered or functionally graded media to enhance practical applicability in geophysical exploration, non-destructive testing, and material characterization.

While the model is validated against classical elasticity theory, future work should include direct comparison with finite-element simulations or boundary-element models, as well as experimental calibration using ultrasonic or seismic data from porous, stressed materials, to further confirm its predictive capability in real-world scenarios.

## Data Availability

The Current submission does not contain the pool data of the manuscript, but the data used in the manuscript will be provided on request.

## Appendix A: Derivation of the void evolution equation

Starting from the linearized constitutive relations for an elastic material with voids^6,7^, the balance equation for the equilibrated stress vector ${h}_{i}$ is given by:

A.1 $${h}_{i,i}+g=\rho \kappa \ddot{\phi },$$

where *g* is the equilibrated body force and *κ* is the inertia coefficient. Under the assumption of negligible body forces and for harmonic wave propagation, this reduces to Eq. (9) in the main text:

A.2 $$\alpha {\nabla }^{2}\phi +\beta {\nabla }^{2}\psi -{\omega}_{0}^{2}\phi =0.$$

Here, *α* and *β* represent stiffness and coupling coefficients related to void distortion, and ${\omega}_{0}$ is a characteristic frequency associated with void dynamics.

## Appendix B: Derivation from equations of motion to wave potentials

This appendix details the steps connecting the equations of motion (13–14) to the wave equations for the potentials (17–20).

- Step 1: Substitute constitutive and strain relations into (13–14) using (10) and (12).
- Step 2: Introduce the Helmholtz decomposition (16) for displacements.
- Step 3: Apply vector identities ($\nabla \cdot (\nabla \times\uppsi )=0$ and $\nabla \times (\nabla \phi )=0$) to separate the equations into irrotational and solenoidal parts.
- Step 4: Collect terms multiplying $\nabla ({\nabla }^{2}\phi )$ and $\nabla \times ({\nabla }^{2}\uppsi )$, respectively. This yields two uncoupled equations:
  $$\left(\lambda +2\mu +P\right){\nabla }^{2}\phi =\rho \ddot{\phi }, (P-wave)$$
  $$\left(\mu -\frac{P}{2}\right){\nabla }^{2}\psi =\rho \ddot{\psi }. (SV-wave)$$

Step 5: Incorporate the void Eq. (15) following a similar potential substitution, leading to the coupled system (17) and (19).

These steps ensure a complete and traceable derivation from the fundamental governing equations to the final wave equations solved in the text.

## Complete derivation of boundary conditions

### Step 1: General traction-free condition

For a free surface at ${x}_{2}=0$ (the horizontal boundary of the half-space), the surface tractions must vanish. In the absence of external forces, this gives:

$${\sigma}_{2j}=0forj=\mathrm{1,2}$$

where ${\sigma}_{2j}$ are the components of the incremental stress tensor at the surface.

Thus, we have two boundary conditions:

B1 $$\begin{array}{cccc}& {\sigma}_{22}=0at{x}_{2}=0& & \end{array}$$

B2 $$\begin{array}{cccc}& {\sigma}_{21}=0at{x}_{2}=0& & \end{array}$$

### Step 2: Constitutive Relations for Initially Stressed Medium

Following Biot’s theory of incremental deformations^4^, for a medium under initial stress, the incremental stress components in plane strain are given by:

B3 $$\begin{array}{cccc}& {\sigma}_{22}=\left(\lambda +2\mu \right){\varepsilon}_{22}+\lambda {\varepsilon}_{11}+P{\varepsilon}_{11}& & \end{array}$$

B4 $$\begin{array}{cccc}& {\sigma}_{21}=2\mu {\varepsilon}_{21}& & \end{array}$$

where:

- $\lambda ,\mu$ are Lamé constants
- $P={\tau}_{22}^{\left(0\right)}-{\tau}_{11}^{\left(0\right)}$ is the initial stress parameter
- ${\varepsilon}_{ij}$ are the infinitesimal strain components

Note: The term $P{\varepsilon}_{11}$ in equation (B3) arises because the initial stress modifies the effective stiffness in the ${x}_{1}$-direction. This is a key feature of Biot’s formulation and appears in classical works^4,5^.

### Step 3: Strain–displacement relations

The infinitesimal strain components are defined as:

$${\varepsilon}_{ij}=\frac{1}{2}\left({u}_{i,j}+{u}_{j,i}\right)$$

For plane strain conditions in the ${x}_{1}{x}_{2}$-plane, we have:

B5 $$\begin{array}{cccc}& {\varepsilon}_{11}=\frac{\partial {u}_{1}}{\partial {x}_{1}}={u}_{\mathrm{1,1}}& & \end{array}$$

B6 $$\begin{array}{cccc}& {\varepsilon}_{22}=\frac{\partial {u}_{2}}{\partial {x}_{2}}={u}_{\mathrm{2,2}}& & \end{array}$$

B7 $$\begin{array}{cccc}& {\varepsilon}_{12}={\varepsilon}_{21}=\frac{1}{2}\left(\frac{\partial {u}_{1}}{\partial {x}_{2}}+\frac{\partial {u}_{2}}{\partial {x}_{1}}\right)=\frac{1}{2}\left({u}_{\mathrm{1,2}}+{u}_{\mathrm{2,1}}\right)& & \end{array}$$

### Step 4: Helmholtz decomposition (Displacement potentials)

To separate compressional (P-wave) and shear (SV-wave) components, we introduce scalar potential *ϕ* and vector potential *ψ* (for the ${x}_{3}$-direction in plane strain):

B8 $$\begin{array}{cccc}& {u}_{1}=\frac{\partial \phi }{\partial {x}_{1}}-\frac{\partial \psi }{\partial {x}_{2}}={\phi}_{,1}-{\psi}_{,2}& & \end{array}$$

B9 $$\begin{array}{cccc}& {u}_{2}=\frac{\partial \phi }{\partial {x}_{2}}+\frac{\partial \psi }{\partial {x}_{1}}={\phi}_{,2}+{\psi}_{,1}& & \end{array}$$

This decomposition ensures:

- ${\nabla }^{2}\phi$ corresponds to irrotational (P-wave) motion
- ${\nabla }^{2}\psi$ corresponds to solenoidal (SV-wave) motion

### Step 5: Express strains in terms of potentials

Substituting (B8)-(B9) into (B5)-(B7):

For $${\varepsilon}_{11}$$:

B10 $$\begin{array}{cccc}& {\varepsilon}_{11}={u}_{\mathrm{1,1}}=\frac{\partial }{\partial {x}_{1}}\left({\phi}_{,1}-{\psi}_{,2}\right)={\phi}_{,11}-{\psi}_{,12}& & \end{array}$$

For $${\varepsilon}_{22}$$:

B11 $$\begin{array}{cccc}& {\varepsilon}_{22}={u}_{\mathrm{2,2}}=\frac{\partial }{\partial {x}_{2}}\left({\phi}_{,2}+{\psi}_{,1}\right)={\phi}_{,22}+{\psi}_{,12}& & \end{array}$$

For $${\varepsilon}_{21}$$:

$$2{\varepsilon}_{21}={u}_{\mathrm{1,2}}+{u}_{\mathrm{2,1}}=\frac{\partial }{\partial {x}_{2}}\left({\phi}_{,1}-{\psi}_{,2}\right)+\frac{\partial }{\partial {x}_{1}}\left({\phi}_{,2}+{\psi}_{,1}\right)$$

$$={\phi}_{,12}-{\psi}_{,22}+{\phi}_{,12}+{\psi}_{,11}$$

B12 $$\begin{array}{cccc}& =2{\phi}_{,12}+{\psi}_{,11}-{\psi}_{,22}& & \end{array}$$

Thus:

B13 $$\begin{array}{cccc}& {\varepsilon}_{21}={\phi}_{,12}+\frac{1}{2}\left({\psi}_{,11}-{\psi}_{,22}\right)& & \end{array}$$

### Step 6: Substitute into stress expressions

**For** $${\sigma}_{22}=0$$ **:**

Substitute (B10) and (B11) into (B3):

$${\sigma}_{22}=\left(\lambda +2\mu \right)\left({\phi}_{,22}+{\psi}_{,12}\right)+\lambda \left({\phi}_{,11}-{\psi}_{,12}\right)+P\left({\phi}_{,11}-{\psi}_{,12}\right)=0$$

Group terms:

$$\left(\lambda +2\mu \right){\phi}_{,22}+\left(\lambda +2\mu \right){\psi}_{,12}+\lambda {\phi}_{,11}-\lambda {\psi}_{,12}+P{\phi}_{,11}-P{\psi}_{,12}=0$$

Separate *ϕ* and *ψ* terms:

$$\left(\lambda +2\mu \right){\phi}_{,22}+\left(\lambda +P\right){\phi}_{,11}+\left[\left(\lambda +2\mu \right)-\lambda -P\right]{\psi}_{,12}=0$$

Simplify the coefficient of ${\psi}_{,12}$:

$$\left(\lambda +2\mu \right)-\lambda -P=2\mu -P$$

Therefore:

B14 $$\begin{array}{cccc}& \overline{)\left(\lambda +2\mu \right){\phi}_{,22}+\left(\lambda +P\right){\phi}_{,11}+\left(2\mu -P\right){\psi}_{,12}=0}& & \end{array}$$

This is Eq. (35) in the manuscript.

## For ${\sigma}_{21}=0$:

Substitute (B13) into (B4):

$${\sigma}_{21}=2\mu \left[{\phi}_{,12}+\frac{1}{2}\left({\psi}_{,11}-{\psi}_{,22}\right)\right]=0$$

Multiply by 1:

$$2\mu {\phi}_{,12}+\mu \left({\psi}_{,11}-{\psi}_{,22}\right)=0$$

Divide by *μ* (assuming $\mu \ne 0$):

15 $$\begin{array}{cccc}& 2{\phi}_{,12}+{\psi}_{,11}-{\psi}_{,22}=0& & \end{array}$$

This is Eq. (36) in the manuscript.

### Step 7: Summary of boundary conditions

At the free surface ${x}_{2}=0$:

35 $$\begin{array}{cccc}& \left(\lambda +2\mu \right)\frac{{\partial }^{2}\phi }{\partial {x}_{2}^{2}}+\left(\lambda +P\right)\frac{{\partial }^{2}\phi }{\partial {x}_{1}^{2}}+\left(2\mu -P\right)\frac{{\partial }^{2}\psi }{\partial {x}_{1}\partial {x}_{2}}=0& & \end{array}$$

36 $$\begin{array}{cccc}& 2\frac{{\partial }^{2}\phi }{\partial {x}_{1}\partial {x}_{2}}+\frac{{\partial }^{2}\psi }{\partial {x}_{1}^{2}}-\frac{{\partial }^{2}\psi }{\partial {x}_{2}^{2}}=0& & \end{array}$$

### Step 8: Key references supporting this derivation

**Table:**

Reference	Contribution
Biot^4^	Foundation of incremental deformation theory; definition of initial stress parameter *P*
Dey & Addy^5^	Application of Biot’s theory to wave reflection problems; derivation of similar boundary conditions
Abd-Alla et al.^15^	Extension to thermoelastic and magnetoelastic media with initial stress
Abo-Dahab et al.^26^	Three-phase-lag thermoelasticity with initial stress and temperature-dependent properties

## Appendix C: Derivation of the traction-free boundary conditions

The general traction-free condition at the surface ${x}_{2}=0$ requires that the incremental stress components vanish:

C.1 $$\begin{array}{cccc}& {\sigma}_{22}=0,{\sigma}_{21}=0at{x}_{2}=0.& & \end{array}$$

For a medium under initial stress $P={\tau}_{22}^{\left(0\right)}-{\tau}_{11}^{\left(0\right)}$, Biot’s theory^4^ provides the following expressions for the incremental stress components in plane strain:

C.2 $$\begin{array}{cccc}& {\sigma}_{22}=\left(\lambda +2\mu \right){\varepsilon}_{22}+\lambda {\varepsilon}_{11}+P{\varepsilon}_{11},& & \end{array}$$

C.3 $$\begin{array}{cccc}& {\sigma}_{21}=2\mu {\varepsilon}_{21}.& & \end{array}$$

The strain–displacement relations are:

C.4 $$\begin{array}{cccc}& {\varepsilon}_{11}={u}_{\mathrm{1,1}},{\varepsilon}_{22}={u}_{\mathrm{2,2}},2{\varepsilon}_{21}={u}_{\mathrm{1,2}}+{u}_{\mathrm{2,1}}.& & \end{array}$$

Using the Helmholtz decomposition, the displacement components are expressed in terms of scalar potential *ϕ* and vector potential *ψ*:

C.5 $$\begin{array}{cccc}& {u}_{1}={\phi}_{,1}-{\psi}_{,2},{u}_{2}={\phi}_{,2}+{\psi}_{,1}.& & \end{array}$$

Substituting (C.5) into (C.4) yields:

C.6 $$\begin{array}{cccc}& {\varepsilon}_{11}={\phi}_{,11}-{\psi}_{,12},{\varepsilon}_{22}={\phi}_{,22}+{\psi}_{,12},2{\varepsilon}_{21}=2{\phi}_{,12}+{\psi}_{,11}-{\psi}_{,22}.& & \end{array}$$

Inserting (C.6) into (C.2) and (C.3) and applying the boundary conditions (C.1) gives:

C.7 $$\begin{array}{cccc}& \left(\lambda +2\mu \right)\left({\phi}_{,22}+{\psi}_{,12}\right)+\lambda \left({\phi}_{,11}-{\psi}_{,12}\right)+P\left({\phi}_{,11}-{\psi}_{,12}\right)=0,& & \end{array}$$

C.8 $$\begin{array}{cccc}& 2\mu \left({\phi}_{,12}+\frac{1}{2}\left({\psi}_{,11}-{\psi}_{,22}\right)\right)=0.& & \end{array}$$

Simplifying (C.7) and (C.8) leads to:

C.9 $$\begin{array}{cccc}& \left(\lambda +2\mu \right){\phi}_{,22}+\left(\lambda +P\right){\phi}_{,11}+\left(2\mu -P\right){\psi}_{,12}=0,& & \end{array}$$

C.10 $$\begin{array}{cccc}& 2{\phi}_{,12}+{\psi}_{,11}-{\psi}_{,22}=0.& & \end{array}$$

Equations (C.9) and (C.10) correspond to Eqs. (35) and (36) in the main text, respectively. This derivation follows the approach established in Biot^4^ and Dey and Addy^5^.

## Abbreviations

${x}_{1},{x}_{2}$: Horizontal and vertical (downward) coordinates
*t*: Time
${u}_{1},2$: Components of displacement vector
*ϕ*: Scalar potential (irrotational part, related to P-waves)
*ψ*: Vector potential component (solenoidal part, related to SV-waves)
${\tau}_{ij}$: Components of the Cauchy stress tensor
${\tau}_{ij}^{\left(0\right)}$: Initial stress components in reference state
*P*: Initial stress parameter: $P={\tau}_{22}^{\left(0\right)}-{\tau}_{11}^{\left(0\right)}$
${\varepsilon}_{ij}$: Infinitesimal strain tensor components
${\omega}_{3}$: Rotation component about *y*-axis
$\lambda ,\mu$: Lamé constants (bulk and shear moduli)
*ρ*: Mass density
*ν*: Poisson’s ratio
*α*: Void stiffness parameter (modifies bulk response due to voids)
*β*: Void-elastic coupling coefficient (couples voids to shear distortion)
${\omega}_{0}$: Characteristic void frequency (intrinsic time scale of void dynamics)
*κ*: Void inertia coefficient (micro-inertial resistance to void change)
*ω*: Angular frequency of incident/reflected waves
*k*: Wave number
${c}_{p}$: Phase velocity of compressional (P) wave
${c}_{s}$: Phase velocity of shear vertical (SV) wave
${\theta}_{0}$: Incident angle of SV-wave (measured from normal)
${\theta}_{1},{\theta}_{2}$: Reflection angles of P-wave and SV-wave
${R}_{c}$: Reflection coefficient of compressional (P) wave (amplitude ratio)
${R}_{r}$: Reflection coefficient of rotational (SV) wave (amplitude ratio)
*∇*: Nabla (gradient) operator
$${\nabla }^{2}$$: Laplacian operator
${}_{,i}$: Partial derivative with respect to coordinate ${x}_{i}$
$\ddot{\left(\cdot\right)}$: Second partial derivative with respect to time
SV-wave: Shear Vertical wave (polarization perpendicular to propagation in ${x}_{1}{x}_{2}$ -plane)
P-wave: Compressional (Primary) wave (polarization parallel to propagation)
3PHL: Three-Phase-Lag (thermoelastic model)
DEM: Discrete Element Method
micro-CT: Micro-Computed Tomography
