# Correction: Elastic wave interaction with a stressed half-space containing voids

**DOI:** 10.1038/s41598-026-64437-4

**Published:** 2026-07-28

**Authors:** S. M. Abo-Dahab, Emad K. Jaradat, Sharif Abu Alrub, Aftab Khan, Hajra Kaneez, E. S. Elidy

**Affiliations:** 1 Mathematics Department, Faculty of Science, South Valley University, Qena, Egypt; 2 Department of Physics, Faculty of Science, Imam Mohammad Ibn Saud Islamic University (IMSIU), 11623 Riyadh, Saudi Arabia; 3 Department of Mathematics, COMSATS, Institute of Information Park Road, Chakshahzad, Islamabad, Pakistan; 4 Department of Mathematics, Faculty of Science, Zagazig University, P.O. Box 44519, Zagazig, Egypt

Correction to: *Scientific Reports* 10.1038/s41598-026-59688-0, published online 10 July 2026

The original version of this Article contained an error in the order of the author names, which was incorrectly given as S. M. Abo-Dahab, Sharif Abu Alrub, Emad K. Jaradat, Aftab Khan, Hajra Kaneez & E. S. Elidy. The correct author order is S. M. Abo-Dahab, Emad K. Jaradat, Sharif Abu Alrub, Aftab Khan, Hajra Kaneez & E. S. Elidy.

The original Article has been corrected.

